# Selection and Arrangement of Teeth, in Constructing Dental Substitutes

**Published:** 1860-08

**Authors:** W. Calvert


### Original Essays and Communications.

### SELECTION AND ARRANGEMENT OF TEETH, IN CONSTRUCTING DENTAL SUBSTITUTES.

BY PROF. W. CALVERT, D. D. S.

IT is a fact, doubtless self-evident to the mind of many in our profession, as well as out of it, that comparatively little regard is had by the mass of practicing dentists to the proper selection and arrangement of teeth.

Individual cases are not unfrequently met with where teeth are inserted, and it may be in point of construction, so far at least as relates to skill in mechanism, faultless, and yet in no particular can they be said to hold a proper analogy to the supplies of nature, or are in that wonted harmony existing in other parts which combine to give character and expression, ever truthful to nature.

In the present age of progress in the arts and sciences, such should not be the case. The professional man who, in the management or treatment of his patients, fails to regard the governing laws of the economy, in securing and preserving the anatomical and physiological relations, is not counted worthy the name to which his profession would entitle him, but is justly reputed as empirical. And can it be considered any the less culpable on the part of the dental practitioner if *he* should ignore, by practice, the first principles of restorative science—a strict adherence to nature? As in the economy of nature, all her complex, systematic and composite arrangements of parts harmonize, so, when invaded by disease, or intercepted by accident, so that the requisition of science and art become necessary, perfect conformity to nature should, as far as practicable, be alone the idea of laudable ambition.

Four points are here severally deserving of special notice, and should claim the attention and consideration of every practitioner, especially those just entering the profession.

No positive indications can here be given by which one may be, unmistakably, guided in the matters under consideration. The naturalist, from his accumulated store of scientific knowledge, is enabled to solve the anatomical problem of the unknown species, having but a single or isolated bone; so the dentist, by careful study, close observation, and experience, should be able to adapt the color, size, and form of the teeth, by the relative analogy they hold to other parts, conformably with each particular age, t mperament, etc.

The first thing, then, to be noticed, is the particular shade of teeth adapted to each case. This, doubtless, is by many considered of but minor importance, and is, therefore, often overlooked, or, at any rate, receives but little of the attention that it deserves or demands. That a distinguishable and even marked difference exists, not only in the color, but in the size and form of the natural teeth of persons of different ages and temperaments, no one will for a moment question; and it will be admitted, as well, that their color changes, from time to time, either from constitutional or other causes, or from advancing age. By the preponderating forces in the human organism, the various temperaments are indicated. To the skin, and to the hair, are imparted their peculiar color; and as by the mysterious processes of nature these are developed, so the teeth, in their normal or abnormal condition, receiving their supplies from the same nutrient source as other parts, under the same controlling influences, in like manner, present common characteristics, and are susceptible of like organic changes, either in the formative processes, or subsequently, after mature development. If, then, we have presented this variety of aspects in color, etc., of the natural organs, we are thus irresistibly led to the urgent necessity of special attention, with regard to a strict conformity to nature, in the insertion of dental substitutes. The inference, then, must be obvious, that we should insist upon the closest possible approximation to nature, first, where we have some of the natural teeth remaining in the mouth with which to compare, and by which to be guided, and, secondly, in suitably appropriating where all of the teeth are wanting—nothing but the individual, age, temperament, etc., to direct.

The size of the teeth is next worthy of note. In this, as well as in other respects, we find the same class of teeth differing in different mouths. In many cases (partial ones) it is by no means difficult to determine upon the size of the teeth required, inasmuch as the position and space to be supplied will sufficiently indicate the same. But, in other cases, the difficulty is increased by the loss of most, if not all of the natural teeth. Here it is especially where proper knowledge and sound judgment combined, are the only safeguards to success.

The form or shape of the teeth also, and especially the front ones, has much to do in securing the most desirable results, as relates to the general and external appearance. Much of the character of a set of teeth depends, indeed, upon this particularly. It is certainly apparent to the mind of any one that no single form, size, or color of teeth is adapted to the general wants; and hence, the implied necessity for proper regard in this particular.

What has just been said, of course, has no reference to the internal, palatal or lingual shapes of the teeth, nor is it my intention to refer to this at present, further than to make this passing allusion.

More, perhaps, than any of the other points spoken of, selfconsidered, has the *arrangement* of the teeth, in their relation to each other, to do with the life-like expression and natural appearance of the mouth. Throughout, in the fitting and arrangement of the teeth upon plates for the mouth, a regular, uniformly set, or symmetrical order should, in all possible cases, be avoided; and, more especially, where nature has not implanted symmetry and beauty in the countenance, featuıes and expression of the individual. Instead of maintaining a perfect order in their arrangement, it will be found, upon trial, that the less the adherence to the ideal of perfection in this respect, (setting one tooth a little high, another low, one out, and another in—in a word, *irregular*), the more pleasing and natural will be the effect in the mouth.

It is almost a universal custom, in the fitting and arrangement of teeth upon plates, to give all of the front ones—both upper and under—an inclination toward the median line, or symphisis. While this is generally in correspondence with nature, as relates to the *upper* teeth, it is not so in regard to the *under* ones. In them (the under) there should be a slight divergence each way from the center,—at the same time setting them quite close together, rather than having the spaces usually separating the upper teeth.

From the variety of age presented, with the contrasted appearance existing in the natural teeth, from youth to middle age, or more advanced life, it becomes apparent that the same general character that is imparted to the denture in youth, is not at all adapted to or befitting the individual beyond the meridian of life. As life advances, instead of the teeth presenting the perfection and beauty of youth, we invariably find their cutting edges and cusps materially abraded, (some more and some less) from their constant attrition, in the fulfillment of their appropriate function.

Now, in the construction of cases, under circumstances so varied by age, there should ever be kept in view this important feature—adaptation to age. Should advanced life be the one involving claim, as the fitting of the teeth goes on, the external cutting edges of the lower, and the internal of the upper ones should be ground off, at such an angle or inclination as will present the abraded edges, cr mechanical abrasion, so characteristic of age.

It is true that the case, with teeth treated thus, so much at variance with common custom, or the usual practice, when examined out of the mouth, may meet the well trained and critical eye unfavorably; yet, when seen, or carefully observed in the mouth, the effect, so produced, will be of the most marked and pleasing kind, greatly contributing to their life-like character.

Much in the expression of the individual countenance depends, also, upon the fullness that is given. In temporary cases, generally, great care is necessary not to overstep the natural limit, and obtain undue fullness, tension of the muscles, and consequent protrusion of the lips. So, likewise, where absorption has taken place, and the deficiency is to be supplied, due regard must be had to the proper contour of the face, so as to attain to the grestest possible natural perfection.

All this, relating as it does to the external, or outward and general appearance, should most unquestionably claim and receive its just share of attention. Still, aside from this, there is another office the teeth have to perform—other than that of an essential, superadded exterior—*that of more appropriate function.* While I would regard, with that attention I esteem due, appearances, in the construction of dental substitutes, I would not be understood, in any way, to divest the subject of its *more* important aspect, in relation to specific function, in which the teeth themselves, the lips, cheeks, and tongue are most intimately concerned—in *prehension*, *mastication*, enunciation, etc.

